# Microglia and synapse interactions: fine tuning neural circuits and candidate molecules

**DOI:** 10.3389/fncel.2013.00070

**Published:** 2013-05-15

**Authors:** Akiko Miyamoto, Hiroaki Wake, Andrew J. Moorhouse, Junichi Nabekura

**Affiliations:** ^1^Division of Homeostatic Development, National Institute for Physiological SciencesOkazaki, Japan; ^2^Department of Physiological Sciences, The Graduate School for Advanced StudyHayama, Japan; ^3^Division of Brain Circuits, National Institute for Basic BiologyOkazaki, Japan; ^4^School of Medical Sciences, The University of New South WalesSydney, NSW Australia; ^5^Core Research for Evolutional Science and Technology, Japan Science and Technology AgencySaitama, Japan

**Keywords:** microglia, synapse, plasticity, elimination

## Abstract

Brain function depends critically on the interactions among the underlying components that comprise neural circuits. This includes coordinated activity in pre-synaptic and postsynaptic neuronal elements, but also in the non-neuronal elements such as glial cells. Microglia are glial cells in the central nervous system (CNS) that have well-known roles in neuronal immune function, responding to infections and brain injury and influencing the progress of neurodegenerative disorders. However, microglia are also surveyors of the healthy brain, continuously extending and retracting their processes and making contacts with pre- and postsynaptic elements of neural circuits, a process that clearly consumes considerable energy. Pruning of synapses during development and in response to injury has also been documented, and we propose that this extensive surveillance of the brain parenchyma in adult healthy brain results in similar “fine-tuning” of neural circuits. A reasonable extension is that a dysfunction of such a homeostatic role of microglia could be a primary cause of neuronal disease. Indeed, neuronal functions including cognition, personality, and information processing are affected by immune status. In this review we focus on the interactions between microglia and synapses, the possible cellular and molecular mechanisms that mediate such contacts, and the possible implications these interactions may have in the fine tuning of neural circuits that is so important for physiological brain function.

## Introduction

Microglia are hematopoietic-cell derived glial cells in the central nervous system (CNS) that function as the only resident immune cells of the CNS (Ginhoux et al., 2010; Prinz and Mildner, 2011). Consistent with their immune cell status, microglia combat brain infections and diseases. This includes playing a significant role in the pathological progression of some of the major neurodegenerative disorders such as Alzheimer's disease, Parkinson's disease, and chronic pain (Finsen et al., 1993; Coull et al., 2005; Hanisch and Kettenmann, 2007; Ransohoff and Perry, 2009; Graeber and Streit, 2010; Kettenmann et al., 2011). Microglia in these disease states are generally secondary responders; in that they undergo a change in morphology and cytokine expression profile in response to the initial insult, through a process defined as an “activation.” Indeed most studies of microglia and disease have focused on the role of these activated microglia in disease progression (Lassmann et al., 1993; Perry et al., 2010). A key remaining question is whether microglia can also be the primary cause of neuronal and psychiatric diseases. To address this, one needs to first characterize their physiological functions in healthy brain and then hypothesis that dysfunction of such physiological roles could result in specific neurological and psychiatric disease.

### Microglia—synapse contacts and functional consequences

Non-“activated” microglia in the healthy brain are highly motile cells, extending and retracting their processes as they survey the microenvironment in the CNS (Nimmerjahn et al., 2005). The multiple components of the synapse represent a major target of this extensive surveillance by the ramified microglia's processes (Wake et al., 2009; Tremblay et al., 2010). In somatosensory cortex of young mice, microglia made brief (≈5 min) contacts with synapses with a frequency of about 1 contact/hour (Wake et al., 2009). Both pre-synaptic boutons and postsynaptic spines were contacted by microglial processes. In visual cortex, the microglia-synapse contacts were examined in closer resolution using 3D reconstruction serial electron microscopy (Tremblay et al., 2010). This study revealed that, in addition to pre- and postsynaptic specializations, microglial processes also contacted peri-synaptic astrocytes and the synaptic cleft (Tremblay et al., 2010).

**Figure 1:**
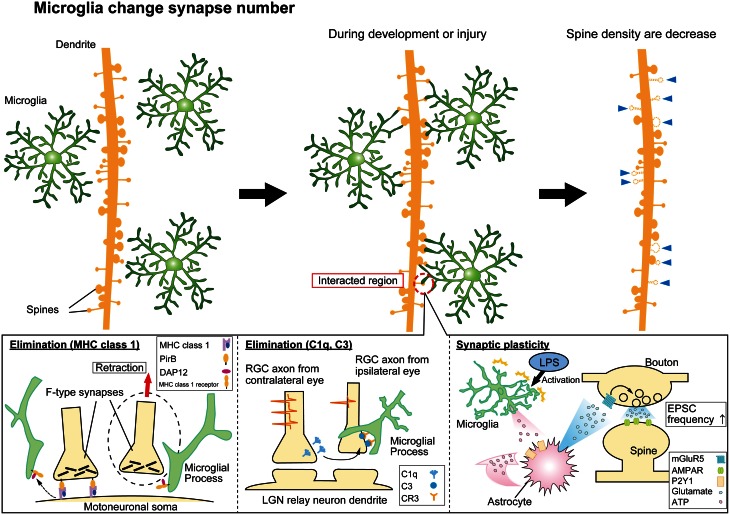
**Synaptic modification by microglial cells.** Microglia monitor and interact with synapses to modulate neural circuit formation and function. Microglia can phagocytose “weak” synapses during development and injury to modify synapse numbers (**top** panels). The lower panels indicate mechanisms mediating functional microglia-synapse interactions. Candidate molecules such as MHC class 1 proteins (**lower left** panel) and complement cascade proteins (**lower center** panel) have been suggested to contribute to the phagocytosis of synapses by microglia. Activated microglia can also modify functional transmission at synapses via signaling pathways involving ATP, astrocytes, and glutamate receptors as indicated (**lower right** panel).

Such a thorough surveillance of all components of the synapse poses the question about what are the functional consequences of this surveillance. In developing and injured brain, a critical function of microglia-synapse contacts appears to be in shaping, or re-wiring, neuronal circuits by phagocytosis (Figure 1). Specific presynaptic (SNAP25) and postsynaptic (PSD95) proteins have been identified inside microglial processes following synaptic contacts, by confocal or immune-gold electron microscopy, respectively (Paolicelli et al., 2011). The synaptic pruning that accompanies developmental refinements of neural circuits coincides with a period of increased density of resident CNS microglia and involves microglial phagocytosis of synapses. This has been most elegantly demonstrated in the developing visual system, where excessive synapses from each retina into the lateral geniculate nuclei (LGN) in the thalamus become pruned by microglia as appropriate binocular visual maps are formed. Genetic deletion of key components of the complement signaling pathway (discussed below) decreased microglial phagocytosis and disrupts this developmental pruning, with LGN neurons inappropriately retaining innervation from both eyes (Schafer et al., 2012). Similarly, conditions which result in enhanced developmental plasticity in V1 cortex of the visual system (dark adaptation during critical period) are associated with evidence for increased phagocytosis of synaptic debris by microglia (Tremblay et al., 2010). Interestingly, microglial contacts with postsynaptic spines appeared to alter their growth and/or morphology (Figure 1), perhaps as part of phagocytosis and remodeling of these elements. In aged animals with reduced auditory function there was also an increase in phagocytotic synaptic debris in microglia in auditory cortex (Tremblay et al., 2012). Hence, developmental and experience-dependent plasticity may involve microglial interactions with synapses and physical remodeling of this component of neural circuits. Experiments to disrupt microglia-synapse contacts in a well-defined synaptic plasticity paradigm and to determine the functional consequences at the ultra-structural and behavioral level will be important in substantiating these exciting hypotheses.

Microglia have long been known to phagocytose neuronal debris as a result of traumatic injury or infections (Perry and Gordon, 1988; Graeber and Streit, 2010), but microglia also can more selectively remove specific neuronal structures, such as neurite extensions in cultured neurons (Linnartz et al., 2012) or axonal terminals and synapses projecting on to injured and degenerating neurons (Oliveira et al., 2004; Yamada et al., 2008). This occurs in the facial injury model, where transection of the motor neurons results in removal of the afferent inputs on to the axotomized neurons (Blinzinger and Kreutzberg, 1968; Graeber et al., 1993). The density of microglia (and astrocytes) increase at the synaptic cleft and appear to tear off the afferent terminals by phagocytosis—the so-called “synaptic stripping” model (Blinzinger and Kreutzberg, 1968). Such synaptic stripping has also been observed in cerebral cortex following microglia activation by bacterial fragments (Trapp et al., 2007). However, as noted by Perry and O'Connor (2010), the close apposition of microglia and synapses in these studies does not constitute evidence for an active role of microglia in “synapse stripping.” In cortex ischemic penumbra, microglia-synapse contacts become markedly prolonged, from ≈5 min to ≈60 min, and this correlates to an increased turnover of presynaptic boutons and postsynaptic spines (Brown et al., 2007; Wake et al., 2009). With prolonged time-lapse imaging, some synapses that had experienced this prolonged contact were indeed observed to disappear, suggesting a possible direct link between microglia-synapse contact and post-ischemic synapse remodeling. Again, experiments designed to selectively modulate these contacts and then to test the effects on post-injury recovery and rewiring will be insightful in determining the functional consequences of such injury induced phagocytosis.

Microglia- synapse interactions are also observed in several neurodegenerative diseases. A reduction in the function and number of synapses, along with an activation of microglia, is an early event in the pathogenesis of Alzheimer's disease, Huntington's disease, and other neurodegenerative diseases (Perry and O'Connor, 2010). Whether microglia initiate such synapse defects is unclear, but two examples of potential mechanisms will be briefly highlighted. In a neuron—microglia co-culture model of Alzheimer's disease, microglia activation releases interleukin-1 that leads to a loss of synaptophysin via phosphorylation of tau (Li et al., 2003). Loss of synaptic proteins like synaptophysin apparently strongly correlates with impaired cognitive function (Coleman et al., 2003; Li et al., 2003). Secondly, the activation of microglial CB2 receptors in a Huntington's disease mouse model (the R6/2 mouse) attenuates neurodegeneration and cognitive decline by reducing microglia activation (Palazuelos et al., 2009). These and similar studies hint at a relationship between microglia activation and synaptic and cognitive defects in neurodegenerative diseases, but further investigation is needed to demonstrate a direct link and underlying mechanisms.

### Mechanisms of microglia-synapse contacts

General observations indicate that there may exist some specific signaling mechanism to direct microglial processes to synapses. Firstly, the surveillance in healthy brain does not seem completely random but is directed toward synaptic compartments (Wake et al., 2009; Tremblay et al., 2012). Similarly in certain situations, such as in developmental pruning and synaptic stripping, microglial phagocytosis is focused on synapses although neuronal soma and other CNS constituents can be frequently phagocytosed by microglia during injury or apoptosis (MaríN-Teva et al., 2004; Sierra et al., 2010). A number of features of synapses—high metabolic rates and activity per unit area, high transmitter and ion turnover, close appositions with astrocytes—may underlie these signals although recent evidence has focused on the importance of neuronal activity. The mechanisms that may mediate microglial-synapse contacts can be broadly classed into “find me” and “eat me” signals, partly based on analogies with the peripheral immune system, and neuronal activity appears to act as a “find-me” signal by both increasing microglial process motility and/or contact frequency (Nimmerjahn et al., 2005; Wake et al., 2009; Tremblay et al., 2010). A recent report from zebrafish larval optic tectum neurons directly correlated a higher relative microglial contact frequency with neurons with higher electrical activity (Li et al., 2012). The model adopted readily enabled the authors to directly visualize neurons and combine imaging of GFP labeled microglia with Ca^2+^ fluorescence as a measure of neuronal activity. More active neurons received more frequent contacts. Transfection of an inward rectifying K^+^ channel (Kir2.1) to reduce global activity (Hua et al., 2005), reduced microglial contact frequency. Furthermore, following contact neuronal activity was decreased, as if microglia were turning down hyperactive neurons (Li et al., 2012). Whether microglial processes are specifically targeted to more active synapses in mammalian CNS has yet to be directly determined and if similar approaches can be applied to mammalian CNS synapses the question may be resolved. The answer is bound to be striking, as the exact opposite is observed for synapse pruning in developing visual system of mice. In developmental synapse elimination, weaker synapses are eliminated while stronger ones are maintained or strengthened, a classic rule that also applies to Hebbian plasticity and learning (Penn et al., 1998; Lichtman and Colman, 2000; Hooks and Chen, 2006; Kano and Hashimoto, 2009). When the activity of afferent inputs from each eyes are decreased by tetrodotoxin, the extent of microglial phagocytosis of their terminals in the LGN is increased. On the contrary: when afferent nerve activity is increased by forskolin injections the extent of microglial phagocytosis is reduced (Schafer et al., 2012). Hence, although neuronal activity may increase microglial motility and/or synapse contact frequency (and neuronal soma contacts in zebrafish), it decreases the extent of phagocytosis of presynaptic terminals at least. How neuronal activity, microglial process dynamics and phagocytosis are linked, and how weaker and stronger synapses are detected and marked for phagocytosis by microglia, are important and fascinating questions that still need to be answered.

Adenosine triphosphate (ATP) has been revealed as a clear “find me” signal, with surveillent microglia strongly attracted to the source of ATP, and undergo activation toward a more phagocytic phenotype, via a signaling pathway involving P2Y purinoceptors (Davalos et al., 2005; Koizumi et al., 2007; Ohsawa and Kohsaka, 2011). ATP is a likely candidate mediating activity-dependent recruitment of microglia (Fontainhas et al., 2011; Li et al., 2012). Glutamate has also been considered a potential candidate “find-me” signal mediating activity dependent microglia migration, although its ability to attract microglia may only hold for a more activated phenotype (Fontainhas et al., 2011). Furthermore, the effects of glutamate may be largely mediated by inducing ATP release from astrocytes, to then recruit and activate microglia (Fontainhas et al., 2011; Wong et al., 2011).

Once microglia are recruited to synapses via a “find-me” signal, one of the potential consequences can be phagocytosis in response to an “eat-me” signal. A number of molecules with analogy to the peripheral immune system have been shown to play some role in triggering phagocytosis of synapses. These include the major histocompatibility complex class 1 group of proteins (MHC-1) and the complement cascade proteins, including C1q and C3 (Figure 1). Genetic disruption of proteins involved in the MHC-1 complex reduces synaptic pruning in development of the LGN synapses (Huh et al., 2000) while increasing the extent of synaptic stripping in the facial nerve axotomy injury model (Oliveira et al., 2004; Cullheim and Thams, 2007). Interestingly, the excess loss of synapses in the injury model is largely accounted for by inhibitory inputs. Investigating how microglia-synapse interactions differ between excitatory and inhibitory synapses is an area that has received very little attention beyond this particular study (Oliveira et al., 2004).

Recent studies showed that the immune complement molecules C1q, C3 and the receptor CR3 (CD11b/CD18) are key molecules contributing to microglial phagocytosis of neurites and synapses (Stevens et al., 2007; Schafer et al., 2012). Retinal ganglion cell (RGC) neurons express C1q, the upstream signaling molecule of C3, at P5 (Stevens et al., 2007). The receptor molecule for C3, CR3 is expressed in microglia (Schafer et al., 2012). Genetic deletion of either the C3 ligand, or the CR3 receptor, reduced inclusions of presynaptic terminals in microglia, indicating a decreased microglial synapse phagocytosis (Schafer et al., 2012). Further details of this interaction were revealed in a hippocampal neuron—microglia co culture model. Removal of the sialic acid cap of the glycocalyx enabled C1q to bind to neurites, “tagging” them for phagocytotic clearance by microglia via C3R (Linnartz et al., 2012). Identifying further these “find me” and “eat me” signals, that undoubtably act in combination with other signaling pathways (including “don't eat me” signals), and whether their role generalizes to other models of plasticity are important areas for future experiments.

### Effects of microglia on synaptic transmission

Interactions between microglia and synapses extends beyond the structural effects in phagocytosing and shaping synapses as described above. Although functional effects have been less well studied, microglia can also clearly influence synaptic transmission and also the functional maturation of synapses.

Pascual et al. (2011) activated microglia in hippocampal slices using bath application of lipopolysaccharide (LPS) and observed an increase in frequency of mEPSPs. A signaling pathway was proposed for this effect based on pharmacological evidence. ATP released by activated microglia then activate astrocytes via P2Y receptors, triggering the release of glutamate which in turn acts on presynaptic metabotropic glutamate receptors on neurons enhancing neurotransmitter release (Figure 1) (Pascual et al., 2011). Peripheral nerve injury resulting in chronic pain also induces microglia to alter synaptic transmission, but via a different signaling pathway. ATP release (likely from both neurons and astrocytes) in the spinal cord stimulates BDNF release from microglia that in turn changes neuronal Cl^−^ homeostasis by decreasing the Cl^−^ efflux transporter, KCC2, and thereby reduces the efficacy of inhibition mediated via GABAergic transmission (Coull et al., 2005; Tsuda et al., 2010).

Microglia may also play some role in maturation of synaptic properties, and this has been suggested by recent studies. Activation of the CX3 chemokine receptor (CX3CR1) by its ligand fractaline (CX3CL1) reduces AMPA-mediated postsynaptic currents via a postsynaptic mechanism (Ragozzino et al., 2006). Recent studies investigating chemokine signaling between neurons and microglia. Paolicelli et al. (2011) examined synaptic activity in the acute hippocampal slice isolated from juvenile mouse lacking the chemokine receptor CX3CR1. In the CA1 of these mice, a transient decrease in microglia density was associated with an impaired phagocytosis of developing synapses, as supported by the increased dendritic spine density. This was associated with altered synaptic properties (enhanced LTD, increased mEPSC frequency in the knockout mouse) consistent with a delayed maturation of excitatory transmission (Paolicelli et al., 2011). A deficiency in LTP in the hippocampal slice has also been recently reported for this CX3CR1 knockout mouse (Rogers et al., 2011). Hoshiko et al. (2012) examined synaptic transmission at thalamo-cortical synapses. Typically, excitatory transmission at these synapses changes its kinetics over development, from a slow EPSC mediated by kainate receptors to a faster EPSC involving AMPA receptors (Kidd and Isaac, 1999; Daw et al., 2007). Changes in NMDA receptor subtypes also contributes to these developmental changes in EPSC kinetics (Carmignoto and Vicini, 1992; Barth and Malenka, 2001; Lu et al., 2001). Hoshiko et al. also used the CX3CR1 knockout mice, reporting that there was a delayed migration of microglia into the cortical layer 4 where these synapses are found, and this was associated with a corresponding delay in the maturation of the AMPA/NMDA ratio. The suggestion was that microglia are also involved in maturation of the postsynaptic receptor subtype at these synapses (Hoshiko et al., 2012).

The above indicate a number of examples by which microglia can interact with synapses, either directly or via associated astrocytes, during developmental plasticity and in response to injury. It will be important to now similarly identify the mechanisms and function of microglia synapses interactions in the healthy adult brain.

## Concluding remarks

In this brief review, we have highlighted some of the recent examples of synapse-microglia interactions and outstanding issues. Microglia play a role in shaping structural features of synaptic connections within neural circuits during development and following injury by phagocytosing pre- and post-synaptic components. Microglial interactions with synapses can also affect the functional maturation of pre- and postsynaptic properties, and influence synaptic transmission in response to injury. The extensive surveillance of all components of synapses by microglia in healthy adult brain, and the modification of this activity by sensory experience and neuronal activity, alludes to key roles of microglia-synapse interactions beyond that just in development and injury. Elucidating the molecular mechanisms and functional significance of these interactions in healthy brain will be crucial for determining whether defects in microglia's physiological function could trigger disease. Already some recent exciting results have implicated microglia as the primary site of defects in Rett's syndrome, a model of autism spectrum disorders (ASD), and Hoxb8-deficiency, a model of obsessive compulsive disorder (Chen et al., 2010; Derecki et al., 2012). Infections and compromised immune system are known risk factors associated with schizophrenia and other psychiatric diseases, which are characterized by alterations in synapse number and function (Glausier and Lewis, 2012). Hence it seems highly plausible to propose alterations in microglia-synapse interactions as a possible cause for a range of neurological disease and we eagerly await further studies to allow this hypothesis to move beyond mere speculation.

### Conflict of interest statement

The authors declare that the research was conducted in the absence of any commercial or financial relationships that could be construed as a potential conflict of interest.
